# Clinical and physiological risk factors contributing to the restricted mobility in older adults: a longitudinal analysis

**DOI:** 10.1186/s12877-024-05230-8

**Published:** 2024-07-24

**Authors:** Xin Jiang, Huiying Tan, Huixia Ren, Huiting Zhou, Jingmei Chen, Zhen Wang, Yi Guo, Junhong Zhou

**Affiliations:** 1 Department of Geriatrics, Shenzhen People’s Hospital, Shenzhen, Guangdong China; 2 The Second Clinical Medical College, Jinan University, Shenzhen, Guangdong China; 3 The First Affiliated Hospital, Southern University of Science and Technology, Shenzhen, Guangdong China; 4 Department of Neurology, Shenzhen People’s Hospital, Shenzhen, Guangdong China; 5 Shenzhen Bay Laboratory, Shenzhen, Guangdong China; 6 Hebrew SeniorLife, Hinda and Arthur Marcus Institute for Aging Research, Roslindale, MA USA; 7 Harvard Medical School, Boston, MA USA

**Keywords:** Mobility restriction, Vascular function, Cognitive-motor performance, Activities of daily living

## Abstract

**Background:**

Mobility limitations (e.g., using wheelchair) have been closely linked to diminished functional independence and quality of life in older adults. The regulation of mobility is pertaining to multiple neurophysiologic and sociodemographic factors. We here aimed to characterize the relationships of these factors to the risk of restricted mobility in older adults.

**Methods:**

In this longitudinal study, 668 older adults with intact mobility at baseline completed the baseline assessments of clinical characteristics, cognitive function, sleep quality, activities of daily living (ADL), walking performance, beat-to-beat blood pressure, and structural MRI of the brain. Then 506 of them (mean age = 70.7 ± 7.5 years) responded to the follow-up interview on the mobility limitation (as defined by if using wheelchair, cane, or walkers, or being disabled and lying on the bed) after 18 ± 3.5 months. Logistic regression analyses were performed to examine the relationships between the baseline characteristics and the follow-up mobility restriction.

**Results:**

At baseline, compared to intact-mobility group (*n* = 475), restricted-mobility group (*n* = 31) were older, with lower score of ADL and the Montreal Cognitive Assessment (MoCA), greater score of Pittsburgh Sleep Quality Index (PSQI), poorer cardio- and cerebral vascular function, and slower walking speeds (ps < 0.05). The logistic regression analysis demonstrated that participants who were with history of falls, uncontrolled-hypertension, and/or greater Fazekas scale (odds ratios (ORs):1.3 ~ 13.9, 95% confidence intervals (CIs) = 1.1 ~ 328.2), walked slower, and/or with lower ADL score (ORs: 0.0026 ~ 0.9; 95%CI: 0.0001 ~ 0.99) at baseline, would have significantly greater risk of restricted mobility (*p* < 0.05; VIFs = 1.2 ~ 1.9).

**Conclusions:**

These findings provide novel profile of potential risk factors, including vascular characteristics, psycho-cognitive and motor performance, for the development of restricted mobility in near future in older adults, ultimately helping the design of appropriate clinical and rehabilitative programs for mobility in this population.

## Background

The capacity to live independently and to function well is important for quality of life and well-being in older adult population [1]. Mobility, defined as the ability to move or walk safely and independently, is the core to such functional independence and critical to everyday activities [2]. Aging and age-related conditions oftentimes induce diminished and even restricted mobility (e.g., the use of wheelchair) in older adults. For example, more than 4.6% of older adults reported the use of wheelchair [3–5] and such ratio increases when turning into older age [5]. These mobility limitations have been closely linked to elevated risk of falls, social isolation, and other health-related events in older adult population [6, 7]. Therefore, it is highly demanded to advance our understanding of the development of such age-related mobility limitations, and the factors contributing to their incidence. This may ultimately help develop appropriate management and rehabilitative strategy for mobility limitations in older adult population.

The maintenance of mobility is dependent upon a complex regulation, consisting of numerous neurophysiological elements, such as the peripheral neuromuscular function enabling appropriate locomotor control [8], supraspinal sensorimotor and cognitive control that is pertaining to the integration of sensory inputs (e.g., visual, somatosensory information) and attention [9], and the cardiovascular regulation (e.g., blood pressure, cerebral blood supply) [10]. The age-related alterations in these underlying elements are associated with the diminished mobility [11] in older adult population. For example, Wakefield et al., showed that older adults who had greater white matter hyperintensities (WMH) at baseline would have greater decline of mobility in next four years [12]. Studies have explored the factors to the mobility difficulties in activities of daily living (e.g., difficulties in using bathroom) [13–18]. However, many of these studies focused primarily on psychological and sociodemographic factors (e.g., social-isolation) [13] to those difficulties, and were based upon the cross-sectional analysis [17] or the analysis focusing on only one factor [15]. The multifactorial contributions to the development of more severe mobility limitations, that is, restricted or even disabled mobility (e.g., the use of wheelchair, lying on the bed due to disability) in older adults with intact baseline mobility have not been explicitly characterized.

In this retrospective analysis, we leveraged the data from a clinical depository consisting of older adults who have the regular physical exam in hospital and completed a series of functional assessments. Multiple clinical and functional characteristics, such as hypertension, WMH, cognitive function, and walking speed, were measured at baseline and the self-reported mobility limitations were then collected after at least one year. Our overarching goal is to characterize the relationships between these clinical and functional factors and the development of restricted mobility in older adults.

## Methods

### Participants

Older adults who completed a clinical visit for routine care at the Department of Geriatrics, Shenzhen People’s Hospital, Shenzhen, China, and expressed interest in participating in the study were recruited. The inclusion criteria were: (1) age ≥ 60 years at the first study visit, and (2) the ability to successfully complete a challenging 30-second walking test without personal/physical assistance [19, 20]. The exclusion criteria were: (1) diagnosis of terminal disease (e.g., cancer) according to their medical record, (2) hospitalization due to any medical procedure (e.g., surgery) within the past 6 months, (3) diagnosis of overt neurological diseases (e.g., dementia, Parkinson’s disease or stroke), (4) chronic kidney disease, (5) heart failure or coronary artery disease, or (6) an inability to understand the study protocol (i.e., cannot answer any two of the three questions related to the study protocol).

All experimental protocols, including the assessments, statistical protocols, were approved by the Institutional Review Board of Shenzhen People’s Hospital in 2020, and retrospectively registered on 06/07/2024 (registration ID: ChiCTR2400085452). It was carried out in accordance with the guidelines of the Declaration of Helsinki. All participants provided written consent prior to participating in any study procedures.

### Study protocol

Older adults first completed one screening visit. On this visit, one study staff introduced the study protocol to the participants, and then they completed a series of questionnaires on the recent clinical information that was not included in their own medical record. Participants then answered three questions on the study protocol to ensure their understanding of the study.

If eligible, two baseline in-person study visits were completed at the Department of Gerontology within the hospital. On the first study visit, eligible participants completed a series of questionnaires to assess demographics, health-related habits (e.g., use of alcohol (i.e., number of drinks taken per week [21]) and smoking [22]), and medication use (e.g., anti-hypertensive medication). Then they completed the assessments including blood pressure assessment, walking performance, cognitive function, and sleep quality. One research team member administrated this visit. On the second study visit, participants completed the structural MRI scan of the brain to assess the WMH. These two visits were each separated by at least 24 h. Throughout the study, Participants were asked to refrain from eating or drinking caffeinated beverages 24 h before and throughout this study.

After at least 12 months from the baseline visits, participants completed the follow-up phone or in-person report of the mobility limitations they experienced. The interval between the date of the second baseline visit and that of the follow-up visit was calculated and included in the following analysis.

### Baseline assessments

#### Demographic information

The demographic information, including age, gender, BMI, and education, was recorded using a self-reported questionnaire on the first baseline study visit.

#### History of falls

Participants were asked if they experienced any falls during the past 12 months. A fall was defined as unintentionally coming to rest on the ground or other lower level, not as a result of an overwhelming external hazard or a major intrinsic event [23]. Those who experienced at least one fall were identified as fallers.

#### Activities of Daily Living (ADL) questionnaire

We used the Chinese version of Barthel Index of activities of daily living questionnaire to assess the functional independence of the participants [24–27]. This questionnaire has been widely used in Chinese populations with great validity and reliability [25–27]. The total score of the ADL was between 1 and 100, and greater score reflected better functional independence in everyday activities [24–27].

#### Montreal Cognitive Assessment (MoCA)

The Chinese version of MoCA was used to assess general cognitive function of each participant [28, 29]. The total score of MoCA (ranging from 0 to 30) was used in the analysis and a lower score reflected poorer cognitive function.

### Sleep quality assessment

The sleep quality was assessed using the Chinese version of Pittsburgh Sleep Quality Index (PSQI) with great validity and reliability in Chinese populations [30–33]. The score of PSQI (ranging from 0 to 21) was used in the following analysis, and greater score of PSQI reflected poorer sleep quality.

### Hypertensive characteristics

The hypertensive status of each participant was extracted from the clinical records and confirmed on the screening visit. Specifically, hypertension was characterized as systolic blood pressure (SBP) ≥ 140 mmHg and/or diastolic BP (DBP) ≥ 90 mmHg at the brachial artery of the left arm using an automated sphygmomanometer. We thus categorized participants into normotensive, controlled-hypertensive (i.e., diagnosis of hypertension with actively taking related medication, yet whose SBP and DBP values were below the diagnostic hypertensive thresholds) and uncontrolled-hypertensive (i.e., participants meeting the hypertensive thresholds of BP whether they were or were not taking anti-hypertension medication). The number of participants in each hypertensive group was then recorded and used in the following analyses.

### BP recordings and BP complexity

Following the previous published protocol, we also measured the continuous beat-to-beat SBP and DBP recordings for at least 10 min [34] on the morning of the first visit by using Finometer PRO system (Finapres Medical Systems B.V., Netherlands) secured to the middle finger of the left hand with the participant lying supine. The SBP and DBP complexity was then calculated using multiscale entropy [35, 36] following the protocols in previous publications [19]. Greater BP complexity has been linked to better vascular functionality and better cognitive-motor performance [34, 37].

### Assessment of arterial stiffness and vascular endothelial function

Arterial stiffness was assessed by measuring the left and right brachial-ankle pulse wave velocity (baPWV) (Omron, Kyoto, Japan). The average baPWV across left and right was then obtained and used in the following analysis [38–40]. Greater baPWV reflected greater arterial stiffness.

The vascular endothelial function was assessed using the flow mediated dilation (FMD) (Unex, Nagoya, Japan) when participants were in a supine position at resting state [40]. Lower FMD reflected worse vascular endothelial function.

### Walking assessment

The walking assessment consisted of two 10-meter trials [41] under each of the following conditions: walking quietly (i.e., single-task) and walking while performing a verbalized serial subtraction of three test (i.e., dual-task). They were asked to walk at their preferred speed along a 10-meter straight hallway. To eliminate the potential influences of acceleration and deceleration during walking on the walking speed measurement, at the beginning of the test, participants were asked to stand on a line labelled on the ground, one meter away from the starting line of the 10 m; and to walk across the end of the test, stopping at the line one meter away from the end. Throughout the walking assessment, participants worn the Mobility Lab™ system (APDM, Portland, OR) consisting of six motion sensors to capture the kinematic data related to walking [42]. In dual-task trials, a three-digit random number was given by the study staff at the beginning of each trial, and the participant was asked to perform the serial subtraction throughout the trial. The walking speed in both conditions, a significant factor to fall risk and cognitive impairment (e.g., dementia) in older adults [43, 44], was then measured. The walking speed of the 10 m in the middle was averaged across two trials within each condition was used in the following analyses.

### MRI and Fazekas scale of white matter hyperintensity

On the second visit, each participant completed the MRI scan of the brain structure consisting of T1, T2 and FLAIR sequences on a 1.5-Tesla MR scanner. The specific parameters of the MRI scan were: T1: repetition time (TR)/ echo time (TE) = 250/2.48ms, slice thickness = 5 mm, echo train length (ETL) = 1, matrix size = 320 × 320 mm; T2: TR/TE = 6000/99ms, slice thickness = 5 mm, ETL = 18, matrix size = 640 × 640 mm; and 3D-FLAIR: TR/TE/inversion time (TI) = 9000/2500/85ms, slice thickness = 5 mm, echo train length (ETL) = 16, matrix size = 512 × 512 mm.

The severity grade of WMH was then assessed separately by two neurologists using the Fazekas scale [45, 46] based upon the brain structural MRI data. Both neurologists were blinded to the demographic and clinical information of the participant. The Fazekas scale divided the white matter into periventricular and deep white matter, and each region was scaled at four grades ranging from 0 to 3 based upon the confluence and size of lesions. If different scales on one brain MRI were given by these two neurologists, a third neurologist was invited. The scale was then discussed and determined by the agreement across all three neurologists. The scale of the whole white matter (i.e., the sum of WMH in periventricular and deep white matter) ranging from 0 to 6 was used in the following analysis. Greater Fazekas scale reflected higher WMH grade.

### Follow-up interview on mobility limitations

During the follow-up assessment, Study staff reached out to the participants or their health proxy via phone or in person. They were asked if they experienced mobility limitations or were with restricted mobility, as defined by the use of wheelchair, cane, or walkers, or be disabled and lying on the bed. Then the participants were categorized into different groups (i.e., intact mobility and restricted mobility) according to their answers.

### Statistical analysis

The statistical analysis was performed using JMP 16. The level of significance was set at *p* < 0.05.

We first categorized participants into two groups, that is, restricted-mobility and intact-mobility group. The normality of the data was examined using the Shapiro–Wilk test, and the homogeneity of variance was examined using the Levene’s test. To compare the baseline demographic, clinical, and functional characteristics between groups, we used one-way ANOVA models when data were normally distributed and the Krusal-Wallis test when the data were not normally distributed for the continuous variables, and chi-square models for the nominal variables.

**Fig. 1:**
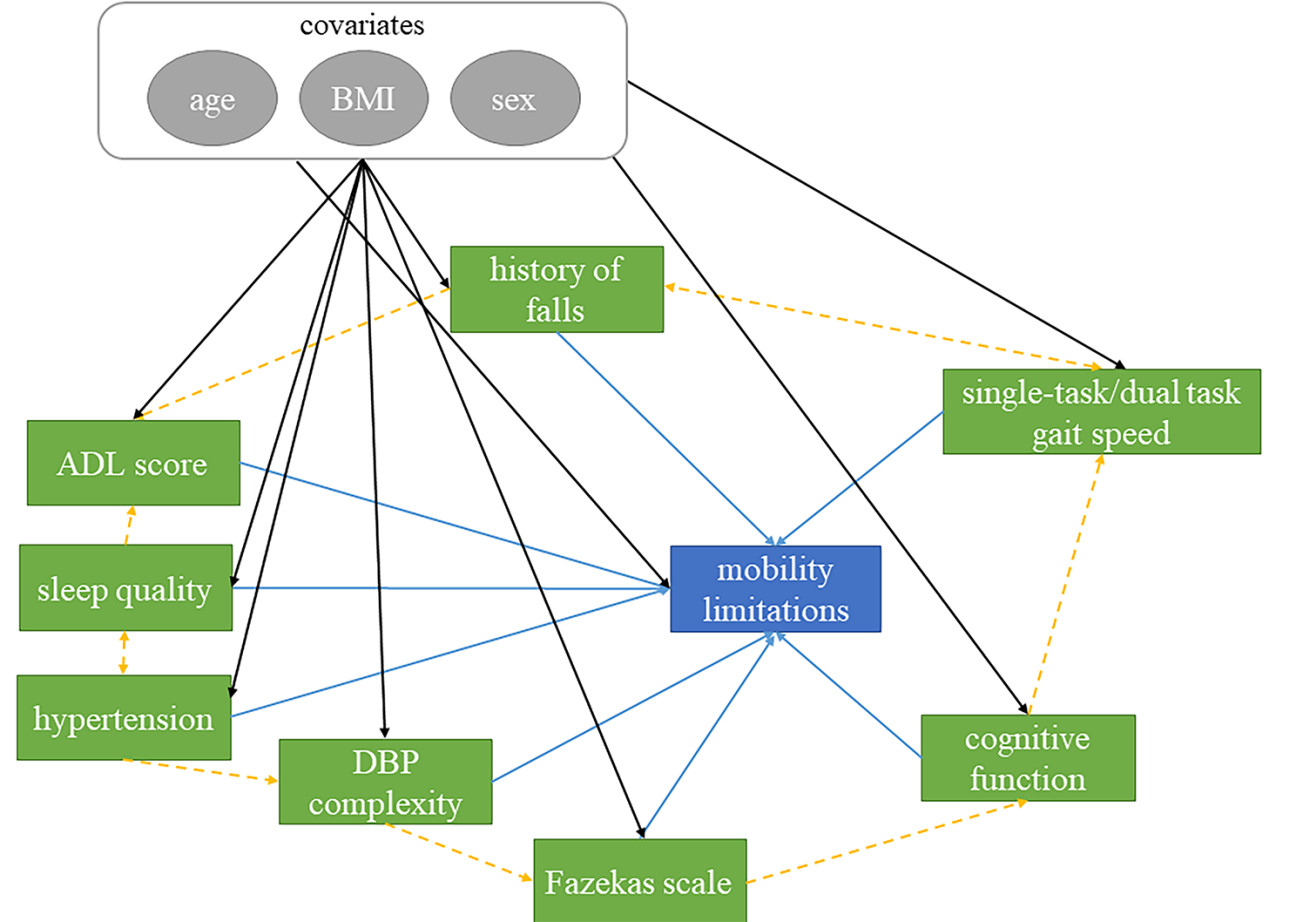
The causal directed acyclic graph (cDAG) informing regression analysis examining the relationships between the clinical, functional and demographic characteristics and the future mobility limitations. The primary outcome/target variable was the mobility limitations as assessed in follow-up (blue square). As we described in Introduction, this outcome was pertaining to many of the assessed clinical, functional and demographic characteristics. In the logistic regression analyses, characteristics (green square) that was significantly different between intact and restricted mobility groups, as determined by the ANOVA models, were the predictor. There is strong evidence indicating that biological age affects numerous underlying neurophysiological systems subserving these characteristics and mobility (e.g., visual acuity, metabolism, etc.). Biological age (grey circles) was therefore designated as a latent variable that captures these unmeasured confounders and included as a covariate in regression models. Additionally, we designated BMI and sex as measured confounders (grey circles), which have been linked to both the predictors and outcome in previous publications. We thus included them as covariates of regression models. Meanwhile, the orange dashed arrow lines indicated the potential relationships between the predictors. For example, the incidence of hypertension may induce diminished BP complexity, indicating alter vascular regulation. Such vascular alteration is associated with the increase in white matter lesions (i.e., greater score of Fazekas scale), and thus poorer cognitive function (lower MoCA score). Therefore, in the multivariate regression model, we carefully examined the potential collinearity between these predictors and excluded those with high collinearity

To examine the relationship between the restricted mobility at follow up and the baseline characteristics, we performed logistic regression analyses. A causal directed acyclic graph (cDAG) (Fig. 1) was used to illustrate the potential relationships between factors, potential confounders and the dependent variable, and to guide the modelling strategy. We first performed the bivariate logistic regression for each potential factor separately. Specifically, the dependent variable was the category of restricted mobility (“intact” or “restricted”), and the model factor was those characteristics with significant differences between intact-mobility and restricted-mobility groups. We calculated odds ratios (OR) and 95% confidence intervals (CI) for each factor of the incidence of restricted mobility. We then used multivariate logistic regression (backward elimination) to explore the relative contributions of the variables to the incidence of restricted mobility and to figure out the best factor profile to mobility limitations. We examined the collinearity among those variables that were significantly associated with mobility limitations in the bivariate analysis (Fig. 1). We complete this by theoretically excluding those that were obviously within the same domain of the function (e.g., single- and dual-task walking). Then remaining variables were included into the multivariate logistic model. Those with significant contributions to the incidence of restricted mobility were identified, and their ORs and corresponding 95% CIs were then obtained. To ensure the collinearity between these variables were acceptable, we calculated the variance inflation factor (VIF) from the regression model. Those with VIF of at least 10 were then removed, and the logistic analyses were reperformed until all the variables in the model were with VIF smaller than 10. Age, gender, BMI, and the interval between baseline and follow-up assessments were included as the covariates in these models.

## Results

**Fig. 2:**
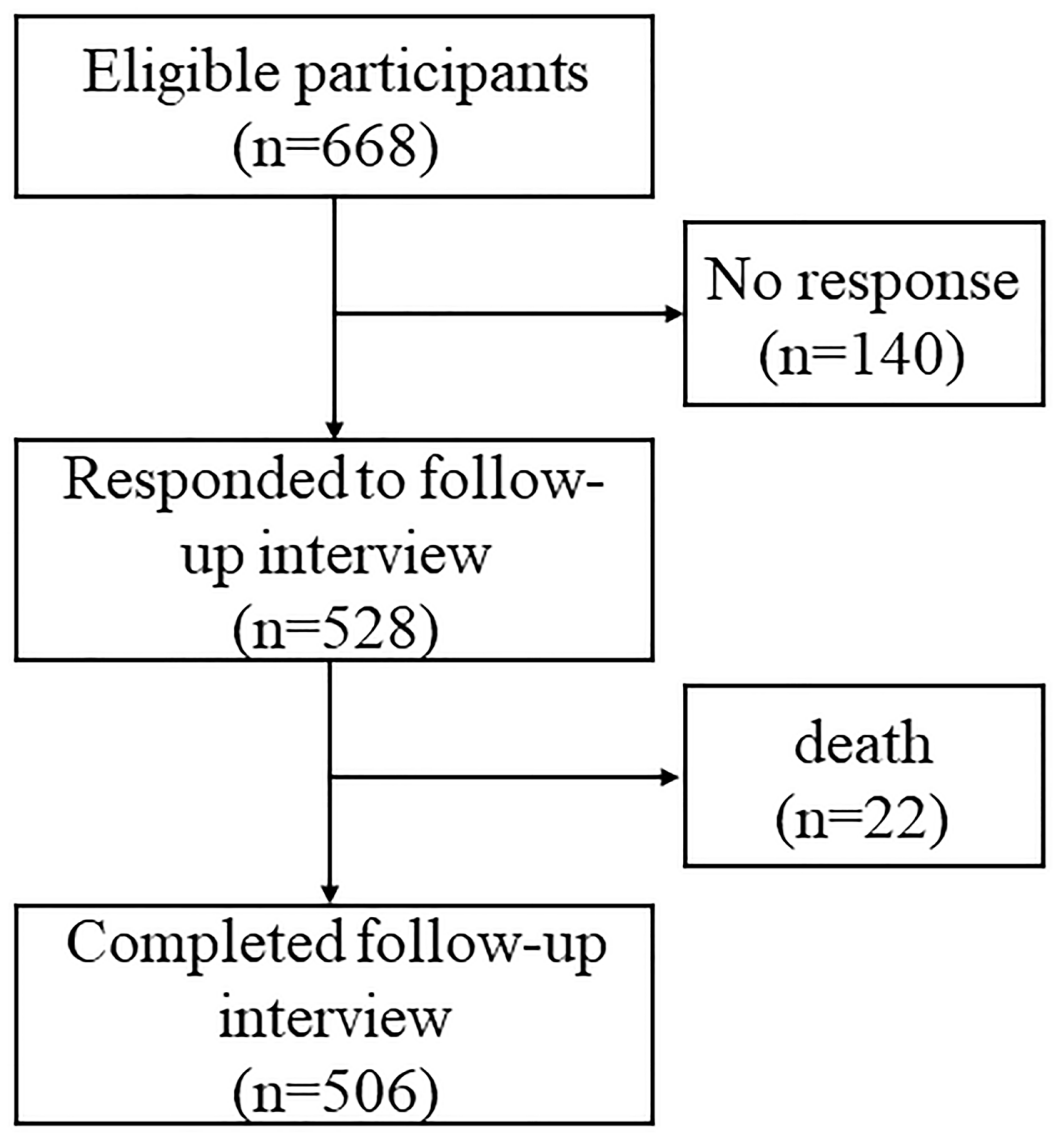
The flowchart of participant enrollment

A total of 668 eligible older adults with intact mobility completed the baseline assessments, and 528 of them responded to the follow-up interview after 18 ± 3.5 months (range: 13 ~ 23 months). Among them, 22 passed away (their family members responded), and the remaining 506 completed the interview (Fig. 2).

### The characteristics between participants with intact and restricted mobility

Table 1 showed the demographic, clinical, and functional characteristics of participants at baseline and of those who completed and did not complete the follow-up interview. At the follow-up interview, 31 (6.1%) participants reported restricted mobility. All the data were normally distributed (ps > 0.55). No significant difference in these characteristics between the follow-up groups and the whole population (*p* = 0.45 ~ 0.88). Compared to those who completed the follow-up interview, those who did not (i.e., non-responder and individuals who passed away) consisted of participants with older age (*p* < 0.01) and greater arterial stiffness (i.e., greater baPWV, *p* < 0.05), and more fallers (*p* < 0.05), but fewer regular alcoholic taker (*p* < 0.05); no significant difference was observed in other characteristics between these two groups (ps = 0.11 ~ 0.88) (Table 1).

**Table 1:** Sociodemographic, clinical, and functional performance in the entire cohort and within each group of mobility

*n* (%) or mean ± SD	baseline (*n* = 668)	lost follow-up (*n* = 162)	follow-up (*n* = 506)			*p* value
			total	intact mobility (*n* = 475)	restricted mobility (*n* = 31)	
age (years)	71.2 ± 7.5	72.6 ± 0.6	70.7 ± 7.5	70.1 ± 7.1	80.9 ± 5.9	**< 0.0001**
Sex (%)						
female	352 (52.7)	81 (46.4)	271 (53.6)	254 (53.5)	17 (54.8)	0.88
male	316 (47.3)	81 (52.6)	235 (47.4)	221 (46.5)	14 (45.2)	
education (years)	9.7 ± 4.7	9.3 ± 0.4	9.9 ± 4.7	9.9 ± 4.7	8.8 ± 5.3	0.18
BMI	24.2 ± 3.8	24.8 ± 3.4	24.4 ± 3.9	24.3 ± 3.9	24.7 ± 3.8	0.61
smoking (n = smokers)	82 (12.5%)	15 (9.2)	67 (13.4)	67 (14.1)	0 (0)	< 0.05
alcohol (n = alcoholic)	40 (6.1%)	4 (2.4)	36 (7.2)	35 (7.4)	1 (3.2)	0.71
ADL	97.2 ± 8.5	96.5 ± 0.7	97.4 ± 8.1	98.0 ± 6.5	88.5 ± 18.5	**< 0.0001**
hypertensive status (n, %=yes)						
normotensive	263 (39.3)	61 (37.6)	202 (39.8)	199 (41.9)	3 (9.7)	**< 0.01**
controlled-hypertension	222 (33.3)	58 (35.8)	164 (32.5)	151 (31.8)	13 (41.9)	
uncontrolled-hypertension	183 (27.4)	43 (26.5)	140 (27.7)	125 (26.3)	15 (48.4)	
diabetes (n, %=yes)	199 (29.8)	43 (26.5)	156 (30.8)	144 (30.3)	12 (38.7)	0.32
chronic pain (n, %=yes)	350 (52.8)	81 (50.0)	269 (53.5)	253 (53.4)	16 (51.6)	0.82
history of falls (n, %=yes)	89 (13.3)	29 (17.9)	60 (11.9)	51 (10.7)	9 (29.1)	**< 0.01**
sleep quality (PSQI score)	9.6 ± 4.7	9.2 ± 0.4	9.7 ± 4.7	9.6 ± 4.7	11.7 ± 5.5	< 0.05
MoCA score	21.5 ± 5.5	20.7 ± 0.5	21.8 ± 5.5	22.0 ± 5.3	16.8 ± 7.9	**< 0.001**
BP complexity						
SBP	1.4 ± 0.3	1.4 ± 0.3	1.4 ± 0.3	1.4 ± 0.3	1.4 ± 0.2	0.36
DBP	1.3 ± 0.3	1.3 ± 0.3	1.3 ± 0.3	1.3 ± 0.3	1.2 ± 0.2	**< 0.05**
baPWV (m/s)	17.9 ± 3.9	18.5 ± 4.6	17.9 ± 3.7	17.6 ± 3.6	21.0 ± 3.6	**< 0.0001**
FMD (%)	3.5 ± 2.1	3.6 ± 2.4	3.4 ± 2.0	3.5 ± 2.0	2.6 ± 1.5	0.07
walking speed (m/s)						
single-task	0.8 ± 0.2	0.8 ± 0.2	0.8 ± 0.2	0.8 ± 0.2	0.6 ± 0.2	**< 0.0001**
dual-task	0.7 ± 0.2	0.7 ± 0.2	0.7 ± 0.2	0.7 ± 0.2	0.5 ± 0.2	**< 0.0001**
score of Fazekas scale	2.5 ± 1.5	2.7 ± 1.4	2.4 ± 1.5	2.4 ± 1.5	3.1 ± 1.5	**< 0.05**

SD = standard deviation. BMI = body mass index. ADL = activities of daily living. PSQI = Pittsburgh Sleep Quality Index. MoCA = Montreal Cognitive Assessment. SBP = systolic blood pressure. DBP = diastolic blood pressure. baPWV = brachial-ankle pulse wave velocity. FMD = flow mediated dilation. The p values are for the comparison between intact and restricted mobility group

As compared to those with intact mobility at follow-up, participants who were with restricted mobility had significantly older age (*p* < 0.0001) but were with similar number of women (*p* = 0.88). BMI (*p* = 0.61), and education background (*p* = 0.18). A significant lower ADL score was observed in restricted-mobility group compared to intact-mobility group (*p* < 0.0001), suggesting poorer functional independence in this group even they were without any mobility limitations at baseline. The restricted-mobility group had no smokers, and only one participant with regular intake of alcohol. The prevalence of diabetes (*p* = 0.32) and chronic pain (0.82) were similar between groups. Compared to intact-mobility group, the restricted-mobility group had significantly greater PSQI score (i.e., poorer sleep quality) (*p* < 0.05) and lower MoCA score (i.e., poorer cognitive function) (*p* < 0.001).

Regarding to vascular characteristics, the prevalence of participants with controlled- and uncontrolled hypertension at baseline were significantly greater in restricted-mobility group than intact-mobility group (*p* < 0.01). Additionally, compared to the intact-mobility group, in restricted group, the DBP complexity (*p* < 0.05), but not SBP complexity (*p* = 0.36), was significantly lower; the average baPWV was significantly greater (i.e., worse arterial stiffness) (*p* < 0.0001); and the score of Fazekas scale was significantly lower (i.e., worse white matter hyperintensities) (*p* < 0.05). A trend toward significant lower FMD (*p* = 0.07) was also observed (i.e., worse endothelial function).

Regarding to the walking performance, compared to intact-mobility group, participants in restricted-mobility group had significantly slower walking speed in both single- (*p* < 0.0001) and dual-task (*p* < 0.0001) conditions.

### The factors associated with restricted mobility: the bivariate logistic analysis

Based upon the results above, we examined the relationships between the scores of ADL, PSQI, and MoCA, history of falls, hypertensive status, DBP complexity, baPWV, single- and dual-task walking speed, Fazekas scale, and the incidence of restricted mobility. Table 2 demonstrated the results of the bivariate logistic analyses. It was observed that each of these included variables was significantly associated with the incidence of restricted mobility. A unit increase in PSQI score (OR: 1.1; 95% CI: 1.0 ~ 1.2), baPWV (OR: 1.002; 95% CI: 1.001 ~ 1.003), and Fazekas scale (ORs: 1.4; 95% CIs: 1.1 ~ 1.8), and/or a unit of decrease in MoCA score (OR: 0.88; 95% CI: 0.81 ~ 0.95), DBP complexity (OR: 0.08; 95% CI: 0.01 ~ 0.4), single- (OR: 0.0023; 95% CI: 00029 ~ 0.02) and dual-task (OR: 0.012; 95% CI: 0.0017 ~ 0.086) walking speed was each associated with a significant increase in the restricted mobility. Particularly, for the history of falls, compared to non-fallers, participants who were fallers had significantly greater risk of being restricted mobility (OR: 3.6; 95%CI: 1.6 ~ 8.3). Similarly, regarding to the hypotensive status, compared to normotensive, individuals who were with controlled-hypertension had significantly greater risk of being restricted mobility (OR: 5.2; 95% CI: 1.4 ~ 18.9), and such risk would further increase for those with uncontrolled hypertension (OR: 8.3; 95% CI: 2.3 ~ 29.1).

**Table 2:** The results of bivariate logistic analysis for the association between each factor and restricted mobility

	OR	95%CI	*p* value
ADL score	0.9	0.91 ~ 0.97	< 0.0001
PSQI score	1.1	1.0 ~ 1.2	< 0.05
MoCA score	0.88	0.81 ~ 0.95	< 0.01
history of falls			
non-fallers	Reference		
fallers	3.6	1.6 ~ 8.3	< 0.01
hypertensive status			
normotensive	Reference		
controlled-hypertension	5.2	1.4 ~ 18.9	< 0.05
uncontrolled-hypertension	8.3	2.3 ~ 29.1	< 0.01
DBP complexity	0.08	0.01 ~ 0.4	< 0.001
baPWV	1.002	1.001 ~ 1.003	< 0.0001
single-task walking speed	0.0023	0.00029 ~ 0.02	< 0.0001
dual-task walking speed	0.012	0.0017 ~ 0.086	< 0.0001
Fazekas scale	1.4	1.1 ~ 1.8	< 0.05

OR = odds ratio. CI = confidence interval. ADL = activities of daily living. MoCA = Montreal Cognitive Assessment. DBP = diastolic blood pressure. baPWV = brachial-ankle pulse wave velocity

Age, gender, BMI, and the interval between baseline and follow-up assessments were included as the covariates

### The contributions of factors to the incidence of restricted mobility: the multivariate logistic analysis

Based upon the results from the bivariate analyses and the cDAG, we performed the multivariate logistic analysis on those with significant association to the incidence of restricted mobility. Considering the overlap between single-task walking speed and dual-task walking speed (*r* = 0.91, *p* < 0.0001), and the important role of cognitive control in dual-task walking speed, which may be captured by MoCA score, we excluded dual-task walking speed in the analysis.

Table 3 showed the results of the multivariate logistic regression analysis, and the variables with significant contributions to the incidence of restricted mobility were identified. Specifically, participants who were a faller (OR: 8.8; 95%CI: 1.1 ~ 70.7), with uncontrolled-hypertension (OR: 13.9; 95%CI: 2.4 ~ 329.2), walked slower in single-task condition (OR: 0.0026; 95%CI: 0.0001 ~ 0.3), with lower ADL score (OR: 0.9; 95%CI: 0.8 ~ 0.99), and/or with greater Fazekas scale (OR: 1.3; 95%CI: 1.1 ~ 1.7) would have significantly greater risk of restricted mobility (ps < 0.05).

**Table 3:** The results of multivariate logistic analysis

	OR	95%CI	*p* value
single-task walking speed	0.0026	0.0001 ~ 0.3	< 0.01
non-fallers (reference)			
fallers	8.8	1.1 ~ 70.7	< 0.05
ADL score	0.9	0.8 ~ 0.99	< 0.05
Normotensive (reference)			
uncontrolled-hypertension	13.9	2.4 ~ 329.2	< 0.05
Fazekas scale	1.3	1.1 ~ 1.7	< 0.05

OR = odds ratio. CI = confidence interval. ADL = activities of daily living

Age, gender, BMI, and the interval between baseline and follow-up assessments were included as the covariates

The VIFs (1.23 ~ 1.89) of these variables were smaller than 10, suggesting acceptable independence between the variables included in this multivariate analysis.

## Discussion

The restricted mobility is a common, but severe condition when people getting into older age, seriously diminishing the functional independence and affecting the quality of life in older adult population. This work explored the associations between the clinical and functional characteristics and the risk of future mobility limitations based upon the data of older adults who have the routine care in a geriatric clinic. As excepted, the results demonstrated that the cardio- and cerebral vascular characteristics, cognitive-motor performance, and functional independence may be associated with the increased risk of restricted mobility, and identified that the history of falls, hypertensive status, walking performance, and cerebral function were the most important factors contributing to the mobility limitations. The knowledge obtained from this work will ultimately inform reshaping the focus of research work and geriatric clinical practice on mobility in older adults.

As expected, the walking performance, level of functional independence, and history of falls are significantly associated with the risk of restricted mobility. The restricted mobility is thought to be one of the most severe consequences of falls and/or fractures, and one significant sign of the loss of functional independence. Our results are consistent with previous studies that the history of falls within a year is an important predictor to future mobility issues (e.g., recurrent falls, etc.). Additionally, the ability to maintain safe walking is the core of mobility [47] in everyday activities. Numerous studies have shown that diminished walking performance in aging process itself is associated with loss of functional independence and oftentimes results in falls in older adult population [48–50]. We here provide confirmatory but novel and critical evidence that even in individuals with intact mobility, their walking performance may be predictive to the risk of mobility limitations in near future.

It is observed here that the characteristics of cardio- and cerebral vascular function, the fundamental of many functionalities in human, are associated with the risk of restricted mobility. Specifically, participants who had hypertension, greater arterial stiffness and/or greater white matter hyperintensities would have greater risk of mobility limitations. This is consistent with previous studies showing that the hypertension, arterial stiffness and while matter hyperintensities are associated with the diminished mobility in older adults [12, 51, 52]. Moreover, we here for the first time, observed that lower complexity of diastolic beat-to-beat blood pressure (i.e., DBP complexity) at baseline is predictive to increased risk of restricted mobility. The bio-neurophysiological systems/procedures of humans, including the blood pressure, are regulated by numerous underlying elements over multiple temporal scales [53]; therefore, their output fluctuation (e.g., beat-to-beat BP series) is complex [54]. Aging and age-related conditions oftentimes alter such complex patterns; and such age-related loss of complexity in those fluctuations, in turn, may capture the subtle changes in the given system pertaining to health-related issues [37]. We and others have recently demonstrated that the complexity metric by quantifying such multiscale dynamics of BP (i.e., BP complexity) is an important characteristic in vascular regulation. For example, the decreased/low BP complexity captures both subtle changes and significant conditions (e.g., hypertension) in the cardiovascular regulation, potentially interfering with the blood supply to the cerebral regions. Such inference may thus contribute to the alterations in structural and functional characteristics in cerebral regions (e.g., increased WMHs), linking to the functional impairments and age-related conditions, including cognitive impairments, diminished walking performance (e.g., slowed walking speed), frailty phenotypes, and the risk of Alzheimer’s disease in older adults [19, 34, 55–57], all of which are related to diminished mobility. We here provide novel and direct evidence that this BP complexity is an independent predictor of the risk of restricted mobility, which thus should be taken into consideration in the clinical routines for the management of mobility in older adults.

It is also observed that poorer cognitive function is associated with increased risk of restricted mobility. This is supported by previous findings on the relationships between cognitive function and mobility. The regulation of mobility is not only dependent upon the musculoskeletal function, but also a host of cognitive function, such as processing speed and attention. Studies have demonstrated that those suffering from cognitive decline (e.g., mild cognitive impairment) had significantly greater risk of mobility-related issues (e.g., slowed walking speed, increased incidence of falls). Additionally, recent research efforts have started to explore the effects of strategies targeting to the enhancement of cognitive function on mobility. Zhou et al., for example, have shown that using transcranial direct current stimulation (tDCS), a non-invasive brain stimulation technique to increase the excitability of cortical prefrontal cognitive regions of the brain can improve walking performance in older adults [58]. These efforts may provide novel directions of targeting cognitive function to help restore mobility in older adults in near future’s rehabilitative practice.

Additionally, poorer sleep quality is also associated with the increased risk of restricted mobility. Sleep problems are common in older adult population [59], and older adults with more complaints of sleep problems had more health-related issues. Several studies have linked poor sleep quality to worse mobility [60–63]. Suzanne et al., showed that, for example, in a group of older women, those who had more sleep problems (e.g., more wakes during sleep) had slower walking speed, longer time to complete chair-stand test and/or poorer grip strength [64]. Taken together, in addition to those factors (e.g., walking performance) that are traditionally focused on, the sleep problems should be considered for the management of mobility, and thus the functional independence in older adults in the near future.

The multivariate logistics regression analysis further suggests that among these important independent predictors, walking speed, history of falls, the level of functional independence, uncontrolled-hypertension, and the white matter lesions (i.e., as assessed by WMH), are the most important factors to the risk of restricted mobility in older adults. These findings may highlight that for the management and rehabilitation of mobility, it is important to track the walking speed, functional independence and the cerebral vascular function by using structural MRI; to use anti-hypertensive medication to control the hypertension; and uniquely, to implement more care for individuals who had a history of falls.

Several limitations should be noted. Though the sample size of this population is large, but the number of participants who reported restricted mobility is relatively small. This may be potentially due to relatively short follow-up duration. Therefore, the observations here should be taken with some cautious. Meanwhile, all the participants were recruited from the hospital setting; and within this population, the responders (who completed follow-up assessment) were younger and with better health status (e.g., less arterial stiffness) as compared to those who lost the follow-up. This potential recruitment and selection bias may affect the generalizability of the observations. Therefore, future studies with longer follow-up period and consisting of other older adult cohorts (e.g., community-dwelling) are needed to examine and confirm the observations here. The variance of the follow-up time is relatively large, though we have included it into our analysis. Future studies to continuously track the rate and timing of restricted mobility and other mobility-related issues, as well as details of the mobility limitations (such as the frequency and the duration of the use of wheelchairs per week, the type of wheelchairs (e.g., motorized) are needed, enabling to perform survival or hazard ratio analysis of mobility limitations, and providing more insights into the relationships between the severity of mobility limitation and the elements underneath its development. We here did not perform follow-up in-person assessments of the clinical and functional performance due to the pandemic; it is thus worthwhile to track these characteristics longitudinally, enabling the characterization of the relationships between the changes of the factors and the risk of restricted mobility. Additionally, we here included sex as the covariate in the analyses. Studies have observed the sex-related differences in mobility in older adults [65, 66]. Therefore, it is worthwhile to explore the influences of sex on the contributions of these factors to the risk of mobility limitations in future studies. Though the collinearity between the factors in the multivariate analysis has been carefully considered (e.g., we did not include dual-task walking speed here, although it is an important metric to characterize the cognitive-motor control, and with an average of 0.1 m/s difference from single-task walking speed [67]) and checked, the relationships between these factors (e.g., Fazekas scale is associated with cognitive function [68, 69]) may contribute to the observation. Therefore, it may be worthwhile to construct models using structural equation modeling to explore the interrelationships more explicitly between these factors and how these interrelationships are associated with the risk of restricted mobility.

## Conclusions

This longitudinal analysis provides novel evidence to the potential risk factors contributing to the development of restricted mobility in a cohort with intact mobility at baseline, including several cardio- and cerebral vascular characteristics, the cognitive-motor performance, and the level of functional independence. The knowledge obtained from this work will ultimately help the design of appropriate clinical routine and rehabilitative programs for mobility in older adults.

## Data Availability

The datasets generated and/or analyzed during the current study are stored and managed in the local data system of Shenzhen People’s Hospital, and are available from the corresponding author on reasonable request.

## Abbreviations

SD: Standard deviation
BMI: Body mass index
ADL: Activities of daily living
PSQI: Pittsburgh Sleep Quality Index
MoCA: Montreal Cognitive Assessment
SBP: Systolic blood pressure
DBP: Diastolic blood pressure
BaPWV: Brachial-ankle pulse wave velocity
FMD: Flow mediated dilation
OR: Odds ratio
CI: Confidence interval
